# Address

**Published:** 1866-04

**Authors:** Geo. H. Cushing

**Affiliations:** Chicago


### ADDRESS.

BY GEO. H. CUSHING, D. D. S., CHICAGO.

Mr. President :—In addressing you at this time upon the subject of "Dental Education," I do not propose to extend my remarks beyond the consideration of a few prominent points, the discussion of which, I conceive, may lead to beneficial results; and shall endeavor, as briefly as possible, to review this matter by considering what are the chief means of "Dental Education; how are those means employed, and how can their efficiency be increased. And whatever of criticism may appear here, will be made in the kindest spirit, with the hope of advancing the cause which we all hold so near our hearts.

The chief means of "Dental Education" would seem to be "the dental colleges," "the dental journals," and "the dental associations."

Our "dental schools" should lay for us, broad and deep, the foundation of our education, in those great principles which must underlie any thorough and scientific "professional education, and though they are not, in all respects, what they should be, and are far from what we all hope to see them; yet they are so much in advance of what we could have expected thirty, or even twenty years ago, that any criticism upon their insufficiency would now be out of place. The chairs of instruction filled, as they are for the most part, by the ablest men in the profession, many of them teachers of long experience and large success as such; while of those younger in years and in experience as teachers, who fill important chairs in some of the colleges, their career as instructors and leaders in the profession, promises to be as brilliant as their ambition could desire. Our colleges demand our most loyal support, and should be nurtured with the tenderest care.

That this 'means' is not employed at all as it should be is too painfully apparent, and this is the fault of the profession. We do not insist half forcibly enough upon the absolute importance of these collegiate courses. The schools are not to blame for this, and we can hardly expect to see *them* taking any great strides in the direction of *internal* reform, while the profession are so slow to avail themselves of the advantages that *are* afforded. I say the profession, because it is the duty of all members of it to see that every student, from their offices, attends the full course of instruction in some of the colleges. This evil *can* be reformed, and it surely *ought* to be.

The reforms which are needed in the colleges themselves are sure to come in good time, as the profession and the public grow up to the proper understanding of what should constitute the true dentist.

Our "dental journals" next claim attention, and the same remarks which were made concerning the schools are, in a great degree, applicable to them.

They have far outstripped our reasonable expectations in the ability with which they are conducted, and in the amount of valuable and instructive matter which they bring monthly or at longer intervals, to every member of the profession, who does his duty by subscribing for them. Yet, do they not fall far short of the good they *might* accomplish? and this, too, through the fault of the profession rather than the conductors of the magazines.

The "journals" are the medium of communication between members of the profession situated at remote points; their columns are open to and earnestly invite the discussion of all matters of interest to the profession, and though much of great value is placed before us now, how much more *might* be, if the profession, generally, were more keenly alive to their duty in this matter? There is not half enough written by members of the profession at large; the contributors to our periodical literature are confined to but a few, while there are hundreds capable of giving instruction by recording their modes of practice, the results of that practice, and their various experience, as well as by presenting theories upon the many points yet to be illuminated, whose pens are ever silent. This should not be so, and it is to be hoped that an improvement in this direction will soon be manifest. Is it not thus evident that we do not employ *this* means of education as we ought; and we are not only, as a profession, open to the above criticisms, but to unqualified censure for not giving sufficient *support* to these "journals?" How many are there who do not take *any* "dental journal?" how many more who do not read one, even at their neighbor's expense? Let a reform be instituted here, for it is most sadly needed.

We come now to consider our "dental associations" as another of the means of education, and perhaps the most valuable of them all. It surely stimulates all other means, for is not the measure of success of our "colleges" and our "journals" in exact ratio to the energy of our various societies, Local, State and National?

In these "associations" we come together with a common purpose, each bringing something that no one else possesses, throwing all into the common stock for the common benefit. Men of various minds, seeing things from various stand points, theorizing differently upon the same subjects, some observing particularly, one class of phenomena, others observing other classes; meeting thus and discussing freely the subjects brought before them, throw light upon a hundred hidden points that otherwise would remain ever obscure. 'Tis from this free interchange of thought and experience that we are enabled to arrive at correct and intelligent theories, upon which all sound practice must be based. Who shall say then if this is not the first in importance of all our educational resources? Prof. Davis says in his work on "Medical Education," "as a basis of all permanent improvement, I must place an efficient internal organization of the profession:" This is certainly as true of the "dental" as of the "medical" profession.

Can we then be too grateful to the few earnest and faithful members of the profession—some of the *most* earnest and faithful of whom are present with us to-day—to whom chiefly is due the credit of inaugurating this associated effort, and of helping it forward by example, by advice and by kind counsel, to its present efficient position as one of the most valuable of our means of instruction? Do we show our gratitude sufficiently by our works? That many of our local societies are in vital activity, and are doing a good work, we have evidence from various sources — in the reports which occasionally appear in the "journals," of their discussions — in the articles which come from the pens of many of their members, and in the character of the men they send to the National association. That all are not thus vigorous I fear is too true, and even with those that show the most vitality, I fear there is a flagging zeal with some of the members, which is unworthy those who claim to be striving in a noble cause. That we do not employ this means as fully as we *might*, is very apparent, though the advance in this direction is more evident than in that of the other means of instruction; yet if the theory of Prof. Davis is correct, that the "colleges are patronized, just in proportion as the importance of the fundamental branches of science become better and more universally appreciated through the medium of these societies," we may reasonably hope soon to see a marked improvement in other directions.

Can not something be done to render this most valuable resource more valuable still? Much may be done if we work in the right spirit, not allowing ourselves to be cast down or discouraged at the obstacles to be overcome, but persistently pressing on, ever looking forward and upward, we shall come at last to a full compensation for all our effort.

Thus far I have spoken of our "local societies;" but I must mention our national association, which might almost claim to be classed by itself as one of the principal means of education which we possess. Conceived in a moment of inspiration by some of our noblest minds, and though inaugurated under discouraging circumstances, yet fostered by their generous care, it has grown to be the valuable instrument that it is in the cause of progress in our profession; and that its usefulness is yet largely to be enhanced can admit of no doubt; but do we employ this means for its largest benefit to the profession now? Is there no suggestion which, if acted upon, would increase the present usefulness of this association? We meet in this association the representative men of the profession; necessarily many of the finest minds, the largest culture, and the most extended experience; but do we get from this auspicious commingling the full advantage which might be expected? Let us look for a moment at the character of some of the discussions. A few of the leading members read papers upon the more abstruse points of physiology and pathology, and a few necessarily monopolize the discussion of these subjects, not only because they are better qualified to do so, but because many who would be glad to take some small part in these discussions, are deterred by the fear of being utterly overwhelmed by the weight of words, which their timidity suggests to them, might follow any humble remark or question they might offer. This is a perfectly natural feeling, (with very modest men at least), and is possessed by many of first rate ability and good acquirements, men who would only need kind and considerate encouragement to render them more actively useful members of the association, and I have sometimes thought that such men were not treated with the consideration which was their due. Then the discussion of these subjects absorbs a very large portion of the time of the association. That the discussion of these subjects is valuable and instructive, no intelligent man would attempt to deny; but do they not take too much time, to the exclusion of perhaps equally valuable subjects? Would not the time be more profitably employed by giving a portion of it to the subject of "practice?" The benefit which would be derived from a more general discussion of this subject, would, I conceive, be very great. There are very many who attend the "national association" who would be glad, indeed, to be instructed by the lips that talk so brilliantly upon these great "basal principles," and who could, if they would, talk equally well upon "practice;" and many, I know, are disappointed that so little is said of a practical character by men eminent and distinguished for their success in the treatment of cases that baffle the skill of the majority of the profession, which might lead them to a better and more intelligent practice in their sphere of action. Gentlemen should not forget that our profession is yet in its infancy—that it is only quite recently that "basal principles" and "scientific knowledge" have come to be regarded as of paramount importance as the foundation of sound and rational practice, and that we can not expect to bring all to the highest standard in a day. The profession is struggling manfully for advancement, is progressing most hopefully, and shall it not be encouraged by every means in our power? Am I wrong in thinking that here, too, a reform is called for? With diffidence, but with all respect, I say that I think this should be remedied. Could these reforms be brought about, then with a "national association" fulfilling its highest mission; with "local societies" in vigorous activity every where; with an increased "periodical literature," more generally contributed to and more generously sustained; and with our "colleges" stimulated to higher excellence in teaching, our profession would march on to the fulfillment of its destiny with unprecedented strides.

I have already occupied more of your time than I proposed to do; but perhaps I may be pardoned for trespassing a little farther upon your patience to consider briefly our position as a profession, as to where we properly belong among the professions.

Much has been said about the duty of the "Medical Profession" to extend to us the right hand of fellowship and to confess our brotherhood. That our profession is properly a branch of the Medical profession, and should be so considered, is beyond dispute. That the basis of our professional education should be thoroughly a medical one, I conceive to be susceptible of demonstration. That we should take rank with the medical profession I fully believe, and that the time will come when we *shall* so stand—unchallenged—is as certain as that this is an age of progress. But when I hear *some* men talk of this matter, and see the efforts they make to obtain the favorable notice of Medical men I can't help thinking that they are in haste to be recognized for what they are not. That there *are* men in our profession at the present time, qualified to stand beside the best in the "Medical" profession, we all know, we see some of them before us to day; but is the profession as a whole, so qualified? We need but to glance at the history and respective ages of the two professions, in order to demonstrate the unreasonableness of this claim. And may we not find some consolation in the thought of the position which the surgical branch of the "medical" profession, held toward the mother profession, so short a time since, as was told us by Prof. Brainerd in his address before the National association, last summer? Now what position more proud than that of the educated surgeon? Surely we may take heart, and "possess our souls in patience," when we see such men as Prof. Brainerd, standing among the leaders of his specialty, both at home and abroad, having his own word for it, that but comparatively a few years ago, the practice of his specialty was entrusted mainly to the hands of barbers; and remembering what he told us too, of the struggle which his branch of the profession had to go through to establish their claims, we should not feel too much discouraged, because we do not at once occupy the place which seems to be ours of right.

We must not forget that we are as yet in a state of development and at an early stage of development too, we cannot expect "to run before we can walk"; but we may rest assured that we shall stand side by side with the "Medical" profession, and shall be recognized by "medical" men, just so soon as we prove ourselves worthy of it—not before; meanwhile let us not be impatient for delayed honors, not envious but emulative, and thus striving with our might, in all honorable ways to advance our standard, we shall come in good time to take our proper place among the liberal professions.

